# Wail of the Second Bicuspid

**Published:** 1886-03

**Authors:** Chas. Jenkins

**Affiliations:** Dresden


### WAIL OF THE SECOND BICUSPID.

BY DR. CHAS. JENKINS, DRESDEN.

Read at the Thirteenth Annual Meeting of the American Dental Society of Europe, held in Berlin, August, 1885.

My acquaintance with your profession began with the attempt of Dr. Tendergreen to regulate my neighbors. This he did at my expense, in the following manner: He set a jack-screw between the right superior cuspid and myself, intending to push the former out. Naturally I was no match for my long-rooted, stubborn antagonist, especially as I was not backed up by the first molar, which had been nearly bored to death, poisoned entirely, pounded to pieces, drawn and quartered by this same practitioner. I was, therefore, pushed out against the cheek about half the distance of my longer diameter. Here was food for reflection, but Dr. Tendergreen was not hungry; he never thought. On graduation at the Dental College he believed that his student-life was already over, instead of just begun. "If I have only sufficient confidence in myself," he said, "I shall succeed." Truth was, self-confidence was the only qualification of which he had a complete outfit.

Without further consideration, he now placed a ligature from the right lateral incisor, which pulled me as far inside the arch as I had previously been outside of it. There he kept me for three months in a position affording space for food, which neither the brush nor the tongue could fully remove. Result, a cavity on my approximal surface. This was filled the next year by Dr. Hammer-Tongs. This gentleman's perfectly sound theory was this: Cohesive gold can be made hard enough to resist successfully all the forces of mastication. Having tested this theory by biting on one of Dr. Hammer-Tongs' fillings, which fell out of my second molar neighbor, I am inclined to regard it as a well-established fact.

But the Doctor's undaunted skill did not prevent a minute cracking of the edge of the enamel at the neck, leaving an invisible interstice above the gold, so that the fluids of the mouth found an open road beneath the filling, whence they burrowed into my vitals, softening the bone down to the pulp chamber. Then came—*Toothache.*

The next day my lady went meekly to Dr. Putty and begged him to relieve her. He had been recommended to her as a gentle operator, who practiced "dentistry without pain." Nor was his reputation entirely undeserved, nor fairly describable by the ill-natured gloss of his rivals, who said that dentistry without pains was a paradoxical phrase. Dr. P. removed the plug, applied oil of cloves, and waited a day or two for the inflammation to subside; then he repeated the operation, this time slightly exposing the pulp. At a third sitting he covered the tender spot with carbolized cork dipped in oxide of zinc, flowed oxy-chloride of zinc upon this cap, and after it had hardened he filled the remainder of the cavity with amalgam. There was slight discomfort, scarcely to be called pain, and a feeling of insecurity for several weeks, during which the pulp being scientifically smothered, died the calm and peaceful death of the righteous; in fact, it died unnoticed.

However, her ladyship soon began to be troubled with neuralgic pains, distributed well nigh impartially over the whole right side of the face. These were imputed by her physicians to several extraordinary causes, till at last one pill-prescriber, less ignorant than the others, passing his finger along the upper row of teeth and discovering a tender spot, advised her to consult Dr. Fumble. The latter diagnosed incipient periostitis, and proceeded at once to open up the right superior second bicuspid. The pulp chamber was found full of septic matter. In one of the canals there was still a live pulp. This Dr. Fumble thought he could save by the capping process, which so far from insuring immunity from pain proved a source of the greatest irritation and distress. He waited till the inflammation reached its height, when he applied arsenic, of which it required three doses to bring matters to a crisis. To further illustrate the beauties of capping, he capped the climax by pushing a broach up this pulp canal and breaking it off, firmly wedged, with the dead matter securely stopped in above it.

I do not need to explain to you what followed this treatment, but one fine morning my lady rose with a face as round as the sun's, and a fit model for the brush of a Dutch painter. She now took especial pains to consult the highest attainable authority, and went to Dr. Cleverfingers, whose skill in manipulation had won him a wide reputation. His firmness and delicacy of touch were in marvellous contrast to all my former experience. He took out the filthy cotton, and with a variety of devices, broaches wrapped in cotton, wisps of bibulous paper, applications of absolute alcohol, etc., made an almost perfect cleansing and disinfection of the canal. The Doctor's patience and thoroughness were only equalled by the patience of the sitter, whose confidence was greatly strengthened by his kind yet firm manner. However, the obstruction in the canal caused him much perplexity. As the canal was thin and wide, he managed to find a passage for a very fine broach by the side of the rusty old intruder inserted by Dr. Fumble. He carried the merest thread of cotton wet in carbolic acid up to the foramen, but the cotton slipped down part way during its progress, and the point of the fine instrument passed through the foramen and remained, as in the case of Dr. Fumble. Seeing that the broach when withdrawn was entirely covered by the fibers, he smiled in triumph. Never a case yet in which, by patient perseverance, he could not reach the foramen. He introduced a paste of iodoform into both canals, and when all signs of irritation had passed and he judged the disinfection to be complete, he proceeded to fill the canals with oxy-chloride of zinc. I am bound to say that the Doctor showed extraordinary skill in this manipulation, and I am also bound to say that he overestimated his success.

He filled the main cavity with gold the next day, and my lady was dismissed with the assurance that all was now done that science could do. That night she slept the sleep of the innocent, but next morning she felt a slight tenderness in the region where so much skill had been bestowed and so many blows had been administered. Delaying, however, to consult the doctor, in the hope for a recovery without help, she was called suddenly to make a journey, and having caught cold the irritation grew steadily worse, so that she was obliged to consult Dr. Dareall, who was in the habit of using heroic remedies, from which marvellous cures were reported. Dr. Dareall attempted to bore out the cement in the nerve canals, and succeeded in forcing his drill to the point of the root—almost—when it pierced the pericementum just below the foramen. The result was not so immediately disastrous as there was reason to expect, and yet this was the last straw that broke my lady's patience. She knew "something had happened," she said, and she would let the tooth decay in her head, or be drawn if it gave pain, rather than consult the profession again. The doctor managed to quiet the pain in the abscessed region, and a fistulous opening having been finally established through the alveolus, he was able to pump through a solution of carbolic acid and glycerine, whereupon he declared the abscess cured. From that day to this the cured abscess has behaved like a small Vesuvius, remaining quiet sometimes for months together, and then most unaccountably resuming activity for a few days or weeks, and then subsiding again for another period of deceitful rest.

But why should I, an old and decayed root, half covered with gum and filled with foul debris, recite any further the wrongs inflicted upon me by your profession. Would it be any use to relate the particulars? Are you not notoriously insensible to the woes you systematically inflict? Just look at the items and sum total and see what constant irritation of the pocket nerve has resulted in my case.

Here are the charges:

| | | |
|---|---|---|
| Dr. H.-T., one double gold filling........ | 30 | marks. |
| Dr. Putty, capping nerve and filling.... | 30 | " |
| Dr. Fumble, treatment and refilling.... | 60 | " |
| Dr. Cleverfingers, " " " .... | 75 | " |
| Dr. Dareall, for curing abscess......... | 60 | " |
| Dr. Pivot, for false crown.............. | 75 | " |
| Dr. Bang, binding together the split root and repivoting.................... | 120 | " |

Total for keeping one tooth in perpetual misery and securing its utter loss in seven years, 450 M., or $1l0. I think from observation that, as bicuspids go, I am only an average case. There is a kind of comfort in that.

